# A novel perspective for M-polynomials to compute molecular descriptors of borophene nanosheet

**DOI:** 10.1038/s41598-023-37637-5

**Published:** 2023-07-25

**Authors:** Rashad Ismail, Annmaria Baby, D. Antony Xavier, Eddith Sarah Varghese, Muhammad Usman Ghani, A. Theertha Nair, Hanen Karamti

**Affiliations:** 1 Department of Mathematics, Faculty of Science and Arts, Muhayl Assir, King Khalid University, Abha, Saudi Arabia; 2 Department of Mathematics, Loyola College, University of Madras, Chennai, India; 3 Institute of Mathematics, Khawaja Fareed University of Engineering & Information Technology, Abu Dhabi Road, 64200 Rahim Yar Khan, Pakistan; 4 Department of Computer Sciences, College of Computer and Information Sciences, Princess Nourah bint Abdulrahman University, P.O.Box 84428, Riyadh, 11671 Saudi Arabia

**Keywords:** Chemistry, Mathematics and computing

## Abstract

Nanomaterials feature exceptional, one-of-a-kind qualities that might be used in electronics, medicine, and other industries. Two-dimensional nanomaterials called borophene have a variety of intriguing characteristics, which helped them to leave an indelible impression in the fields of chemistry, material science, nanotechnology, and condensed matter physics. The concept of modelling the structure of a molecule or chemical network to a chemical graph and then quantitatively analysing them with the aid of topological descriptors was a major advance in the fields of mathematics and chemistry, with a wide range of applications. M-polynomial approach is a very versatile and quick method for computing the degree-based descriptors of chemical graphs or networks. The degree-based descriptors of the $\beta _{12}$-Borophene nanosheet are established in this study utilising the M-polynomial technique. A program code that enables to generate the M-polynomial of any chemical structure was developed in Java platform and the same is displayed. At the conclusion, the numerical and graphical comparison based on the identified analytic expressions is also provided. Additionally, the QSPR analysis was also carried out and the outcoms are presented therein.

## Introduction

The discovery of the two dimensional nanomaterial, graphene bought a stupendous research zest in the field of two dimensional materials. It has led to a remarkable response in the material field and appreciably improved the applications of two dimensional materials in various fields. The unique and superlative properties, the outstanding performances and potential applications of these materials have grabbed major attention from the scientific experts. Two dimensional nanosheets are ultrathin compared to bulk materials and have majority of atoms exposed to the surface, so that it can have greater surface areas, higher chemical and physical activity and quantum confinement effects^1^. This nature enrich them with special photonic, electronic, catalytic, and magnetic properties. Also these nanomaterials have found great application in bio-like materials, drug carriers, biosensors, electronic devices, etc.

Among the two-dimensional allotropes of boron, borophene offers a variety of intriguing characteristics. Due to its minimal weight, borophene has exceptional mechanical stiffness and superconducting characteristics. It also possesses a number of exceptional therapeutic features, which expands the scope of its application in the medical and technological sectors. Owing to its remarkable structural endurance and high reliability, borophene has the potential to be used as a channel for drug distribution in immunotherapy and is predicted to have strong immunological function and catalytic activity^1^. These nanoparticles, with their exceptional physical and chemical attributes are ideal for a wide range of scientific and technological purposes, including higher energy fuel, elevated temperature devices, coatings, atomic engineering, and atmospheric emissions. In the areas of chemistry, bioengineering, nanoelectronics, and quantum physics, borophene has steadily gained significance. In contrast to graphene, borophene exhibits a triangular framework with hexagonal cavities (HH), and the distribution of these cavities changes over the entire structure. $\beta _{12}$, $\chi ^3$, $2-Pmmn$ and honeycomb are the most researched borophene nanostructures. While the $\beta _{12}$ and $\chi ^3$ variants of borophene have planar configurations devoid of vertical oscillations and various patterns of systematic boron voids, the $2-Pmmn$ phase of borophene has a corrugated structure, while the honeycomb borophene seems to have a structural resemblance to graphene^2^.

Graph theory, a sub-branch of discrete mathematics deals with the study of connection between things. This study was extended to chemical structures and networks paving way to the concept of chemical graph theory. Thus combining graph theory and chemistry, another sub-branch, chemical graph theory emerged, which utilised the concept of graph theory to characterize molecular structures^3^. Here the idea is to model the structure of a chemical compound or networks to chemical graph and is further analyzed quantitatively with the help of topological descriptors. Topological descriptors have found application in the field of chemical graph theory and are crucial in study of structural properties with many applications in structural chemistry. The IUPAC^4^ defines the topological index as “A topological index is a numerical value which is associated with the chemical constitution and is used for correlation of chemical structure with various physical properties, chemical reactivity, or biological activity”. Topological descriptors help in a very appealing way to determe the physical, chemical, biological or pharmacological properties of a chemical structure. It has application in vast areas of chemistry, informatics, biology, Quantitative Structure-Property Relationships (QSPR) and Quantitative Structure-Activity Relationships (QSAR), etc.^3, 5–9^. Wiener index is the first known structural descriptor, introduced by H. Wiener in 1947^5^. Using the concept, Wiener defined the boiling points for alkanes. Subsequently, in 1972, Gutman and Trinajstić^10^ introduced the first and second Zagreb indices. Further, many modified forms and variants of Zagreb indices were introduced, such as modified Zagreb index^11^, Augmented Zagreb index^12^ etc. In the study of heat formation of heptanes and octanes, the augmented Zagreb index is useful. In 1975, Randić proposed the Randić index^8^, which has application in the field of drug design^13^. A generalized version of Randić index^14^ was also defined. Another variation of the Randić index, which is known as Harmonic index was first introduced and defined by Siemion Fajtlowicz^15^. In chemical graph theory, there are many more topological descriptors, especially many degree based descriptors. The application of these structural descriptors in various fields are remarkable^16^ and researchers working on this concept are coming up with new descriptors that correlates with the structural properties of a chemical structure more precisely^17, 18^.

Traditionally, the structural descriptors were computed directly using the definitions, which are time consuming. Renowned researchers established several techniques to compute the structural descriptors^19–21^, inorder to save computational time. One among them was the polynomial representation of structural descriptors, which acquired broad attention and acceptance in the literature^22^. To compute wiener index, the Hosoya polynomial, which is also known as Wiener polynomial^23, 24^ was used. It was also useful in the computation of Hosoya index, Hyper Wiener index etc. The wiener index is obtained by evaluating the derivative of Hosoya polynomial by equating the variable to 1. Later, in 2015, the concept of M-polynomial was proposed by Deutsch and Klavžar^25^. The concept of M-polynomial is to provide a general polynomial with the help of which, one can determine various degree based structural descriptors.

**Figure 1:**
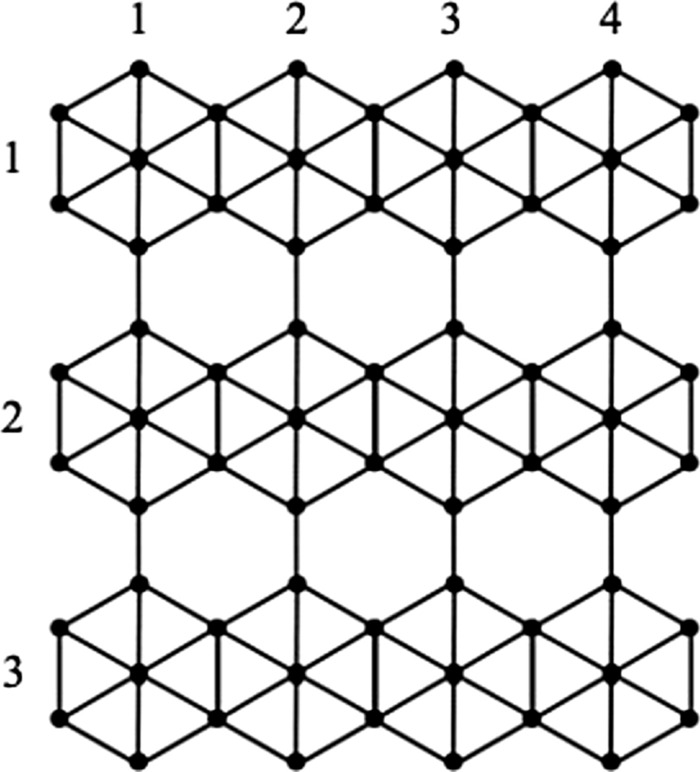
$\beta _{12}$-Borophene nanosheet, $\beta _{12}-BN(r,c)$, where $r=3$ and $c=4$.

In this work, various degree based descriptors of $\beta _{12}$-Borophene nanosheet^2^ are computed using M-polynomial approach. The structural descriptors of borophene nanosheets and borophene nanotubes of other variations, namely, $2-Pmmn$, $\chi ^3$ and honeycomb were studied by researchers^26–28^. In this paper, the $\beta _{12}$-Borophene nanosheet is denoted as $\beta _{12}-BN(r,c)$, where *r* is the count of rows and *c* is the count of columns that hexagons with centre vertex are repeated. The molecular graph of $\beta _{12}-BN(r,c)$ with *r* taking value 3 and *c* taking value 4 is can be denoted as $\beta _{12}-BN(3,4)$, and the same is presented in Fig. 1.

## Mathematical concepts

This section comprises of the notations and the concepts that are employed for the study. Consider a simple graph $\Omega$, which is connected. The vertex set is denoted by $V(\Omega )$ and $E(\Omega )$ denotes the edge set of $\Omega$. The degree of a vertex $\mu \in V(\Omega )$ is denoted as $d_{\mu }$, which is the number of edges incident to the vertex.

The first Zagreb index and second Zagreb index^10^ are defined as,

$$\begin{aligned} M_1 (\Omega )=\displaystyle \sum _{\mu \eta \in E(\Omega )}( d_{\mu }+d_{\eta }), \quad M_2 (\Omega )=\displaystyle \sum _{\mu \eta \in E(\Omega )}( d_{\mu }\times d_{\eta })\,\, \text {respectively.} \end{aligned}$$

These indices are recogonised to provide quantitative measures of molecular branching^10^ and even used in the study of complexity of chemical compounds^16^.

Researchers have developed modified versions Zagreb indices and among them two are the second modified Zagreb index^11^, $M_2^m (\Omega )$ and augmented Zagreb index^12^, $A(\Omega )$.

$$\begin{aligned} M_2^m (\Omega )=\displaystyle \sum _{\mu \eta \in E(\Omega )}\frac{1}{ d_{\mu } \times d_{\eta }}, \quad A(\Omega )=\displaystyle \sum _{\mu \eta \in E(\Omega )} \bigg (\frac{d_{\mu } \times d_{\eta }}{d_{\mu } + d_{\eta }-2}\bigg )^3. \end{aligned}$$

In the line of Zagreb indices, the eminent scientist Randić proposed the Randić index^8^,

$$\begin{aligned} R(\Omega )=\displaystyle \sum _{\mu \eta \in E(\Omega )} {(d_{\mu }\times d_{\eta })^{-1/2}}. \end{aligned}$$

Later a generalized version of the Randić index was introduced, replacing $(-\frac{1}{2})$ by a fixed number $\kappa \in \mathbb {R}$, known as the general Randić index^14^ given by, $R_{\kappa }(\Omega )=\displaystyle \sum _{\mu \eta \in E(\Omega )}(d_{\mu }\times d_{\eta })^{\kappa }$.

The general reciprocal Randić index^29^ is defined as $RR_{\kappa }(\Omega )=\displaystyle \sum _{\mu \eta \in E(\Omega )}\frac{1}{(d_{\mu }\times d_{\eta })^{\kappa }}$. When $\kappa =-\frac{1}{2}$, the reciprocal Randć index is obtained.

Harmonic index^15^ is an another version of Randić index given by, $H(\Omega )=\displaystyle \sum _{\mu \eta \in E(\Omega )}\frac{2}{d_{\mu }+d_{\eta }}$.

The hyper Zagreb index^30^ and the forgotten index^31^ (also known as F-index) are defined as,

$$\begin{aligned} HM(\Omega )=\displaystyle \sum _{\mu \eta \in E(\Omega )}(d_{\mu }+d_{\eta })^2, \quad F(\Omega )=\displaystyle \sum _{\mu \eta \in E(\Omega )}\big ({d_{\mu }}^2+{d_{\eta }}^2\big ). \end{aligned}$$

One of the major application of F-index is that it is found to be appropriate for testing the properties of drug related molecular structures^31^.

The sigma index^32^ is given by, $\sigma (\Omega )=\displaystyle \sum _{\mu \eta \in E(\Omega )}(d_{\mu }-d_{\eta })^2$.

To improve the Quantitative Structure-Activity-Property Relationships studies, Vukičević and Gašperov^33^ established a new class of structural descriptors, called as “discrete Adriatic indices” and it comprises of one hundred and forty-eight descriptors. The symmetric division index^34^ is one among them and is a significant predictor of physicochemical properties of chemical compounds. It is also noted that, among all the existing structural descriptors, the symmetric division index has the strongest correlation for predicting the total surface area of polychlorobiphenyls^33^. It is given as, $SDD(\Omega )=\displaystyle \sum _{\mu \eta \in E(\Omega )} \frac{{d_ {\mu }}^2+{d_{\eta }}^2}{d_{\mu } \times d_{\eta }}$.

The inverse sum index^35^, is defined as $I(\Omega )=\displaystyle \sum _{\mu \eta \in E(\Omega )} \frac{d_ {\mu } \times d_{\eta }}{d_{\mu } + d_{\eta }}$.

The atom bond connectivity index is utilized in theoretical chemistry for designing chemical compounds with specific physicochemical properties or given pharmacological and biological activities. The geometric arithmetic index and sum-connectivity index are also correlated with the physical and chemical properties of chemical compounds and used in QSPR and QSAR studies. The atom bond connectivity index^36^, $ABC(\Omega )$, geometric arithmetic index^37^, $GA(\Omega )$ and sum-connectivity index^38^, $SC(\Omega )$ are defined as, $ABC(\Omega )=\displaystyle \sum _{\mu \eta \in E(\Omega )}\sqrt{\frac{{d_ {\mu }+d_{\eta }-2}}{{d_{\mu } \times d_{\eta }}}}$, $GA(\Omega )=\displaystyle \sum _{\mu \eta \in E(\Omega )}\frac{2\sqrt{d_{\mu } \times d_{\eta }}}{d_{\mu } + d_{\eta }}$ and $SC(\Omega )=\displaystyle \sum _{\mu \eta \in E(\Omega )}\frac{1}{\sqrt{d_ {\mu }+d_{\eta }}}$.

For $\Omega$ be a graph and $m_{ij}(\Omega ),i,j \ge 1$ be the number of edges $\mu \eta \in E(\Omega )$ such that $(d_{\mu } , d_{\eta }) = (i , j)$, the M-polynomial is depicted as^25^,

$$\begin{aligned} M(\Omega ;g,h)=\displaystyle \sum _{i\le j}m_{ij}(\Omega ) \quad g^i h^j. \end{aligned}$$

The following operators are required while computing the degree based descriptors using the M-polynomial concept^25^.

$$\begin{aligned}&D_g(t(g,h))=g \quad \frac{\partial {t(g,h)}}{\partial {g}}, \quad D_h(t(g,h))=h \quad \frac{\partial {t(g,h)}}{\partial {h}}\\&S_g(t(g,h))= \int _{0}^{g} \frac{t(f,h)}{f} \,df, \quad S_h(t(g,h))= \int _{0}^{h} \frac{t(g,f)}{f} \,df\\&J(t(g,h))= t(g,g), \quad Q_{\alpha }(t(g,h))= g^{\alpha } \quad t(g,h); \alpha \ne 0. \end{aligned}$$

The formulas for deriving the degree based structural descriptors from M-polynomials are given in Table 1^22, 25^.

**Table 1:** The formulas for deriving the degree based descriptors from M-polynomials.

Degree based descriptor	*t*(*g*, *h*)	Derivation from $M(\Omega ;g,h)$
First Zagreb	$g+h$	$(D_g+D_h)(M(\Omega ;g,h))|_{g=h=1}$
Second Zagreb	*gh*	$D_g D_h(M(\Omega ;g,h))|_{g=h=1}$
Second modified Zagreb	$\frac{1}{gh}$	$S_g S_h(M(\Omega ;g,h))|_{g=h=1}$
Hyper Zagreb	$(g+h)^2$	$(D_g+D_h)^2(M(\Omega ;g,h))|_{g=h=1}$
Harmonic	$\frac{2}{g+h}$	$2 S_g J(M(\Omega ;g,h))|_{g=1}$
General Randić	$(gh)^{\kappa }$	${D_g}^{\kappa } {D_h}^{\kappa }(M(\Omega ;g,h))|_{g=h=1}$
General reciprocal Randić	$\frac{1}{(gh)^{\kappa }}$	${S_g}^{\kappa } {S_h}^{\kappa }(M(\Omega ;g,h))|_{g=h=1}$
Forgotten	$g^2+h^2$	$({D_g}^2+{D_h}^2)(M(\Omega ;g,h))|_{g=h=1}$
Symmetric division	$\frac{g^2+h^2}{gh}$	$(D_g S_h+D_h S_g)(M(\Omega ;g,h))|_{g=h=1}$
Inverse sum	$\frac{gh}{g+h}$	$S_g J D_g D_h(M(\Omega ;g,h))|_{g=1}$
Sigma	$(g-h)^2$	$(D_g-D_h)^2(M(\Omega ;g,h))|_{g=h=1}$
Augmented Zagreb	$\big (\frac{gh}{g+h-2}\big )^3$	${S_g}^3 Q_{-2} J {D_g}^3 {D_h}^3(M(\Omega ;g,h))|_{g=1}$

## M-Polynomial

This section comprise of the direct computation method of determining the M-polynomial for the proposed structure and a progam code that generates the M-polynomial of any chemical graph.

### M-Polynomial of $\beta _{12}$-borophene nanosheet

#### Theorem 1

Let $\Omega =\beta _{12} - BN(r,c); r, c\ge 1$ be the chemical graph of the $\beta _{12}$ - borophene nanosheet as seen in Fig. 1, then it’s M-polynomial is

$$\begin{aligned} M(\Omega ;g,h)=&(2r+4)g^3 h^3 +(4r-4)g^3 h^4 +(4c-4)g^3 h^5 +(4r+2c) g^3 h^6 +(cr-c)g^4 h^4\\ {}&+4(r-1)(c-1)g^4 h^5 +(2cr-2c)g^4 h^6 +(cr-r)g^5 h^5 +(4cr-4r)g^5 h^6. \end{aligned}$$

#### Proof

**Figure 2:**
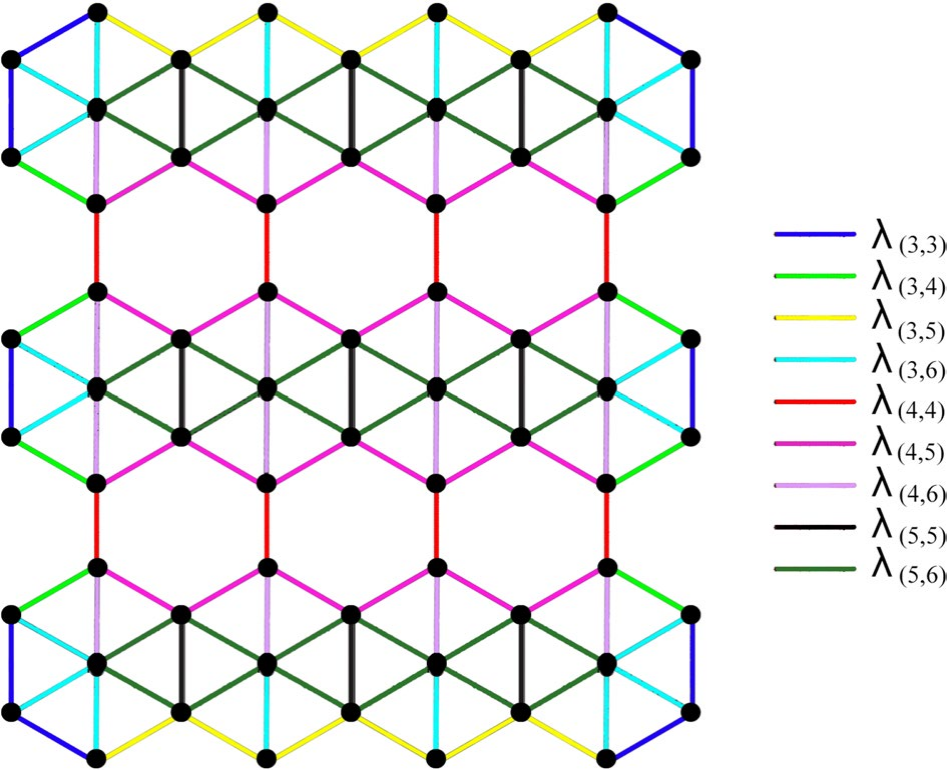
Edge partitions of $\beta _{12}$-Borophene Nanosheet, $\beta _{12}-BN(3,4)$.

Let $\Omega =\beta _{12} - BN(r,c); r, c\ge 1$. $\Omega$ has $5rc+2r$ vertices and $(12r-1)c+r$ edges. The edge set $E(\Omega )$ can be partitioned into sets $\lambda _{(i,j)}=\{(\mu ,\eta )\in E(\Omega ): d_{\mu }=i, d_{\eta }=j\}$, based on the vertex degree and by observation, clearly it can be said that there are nine types of edge partitions, say $\lambda _{(3,3)}$, $\lambda _{(3,4)}$, $\lambda _{(3,5)}$, $\lambda _{(3,6)}$, $\lambda _{(4,4)}$, $\lambda _{(4,5)}$, $\lambda _{(4,6)}$, $\lambda _{(5,5)}$ and $\lambda _{(5,6)}$ for *G*. The edge partitions for $\beta _{12} - BN(3,4)$ are shown in Fig. 2.

It can be also observed that

$$|\lambda _{(3,3)}|=2r+4$$; $$|\lambda _{(3,4)}|=4r-4$$; $$|\lambda _{(3,5)}|=4c-4$$;

$$|\lambda _{(3,6)}|=4r+2c$$; $$|\lambda _{(4,4)}|=rc-c$$; $$|\lambda _{(4,5)}|=4(r-1)(c-1)$$;

$$|\lambda _{(4,6)}|=2c(r-1)$$; $$|\lambda _{(5,5)}|=rc-r$$; $$|\lambda _{(5,6)}|=4r(c-1)$$.

By definition of M-polynomial,

$$\begin{aligned} M(\Omega ;g,h)=&\displaystyle \sum _{i\le j}m_{ij}(\Omega ) \hspace{0.1cm} g^i h^j. \end{aligned}$$

Therefore,

$$\begin{aligned} M(\Omega ;g,h)=&\displaystyle \sum _{3\le 3}m_{33}(\Omega ) g^3 h^3 +\displaystyle \sum _{3\le 4}m_{34}(\Omega ) \quad g^3 h^4 +\displaystyle \sum _{3\le 5}m_{35}(\Omega ) \quad g^3 h^5\\ {}&+\displaystyle \sum _{3\le 6}m_{36}(\Omega ) \quad g^3 h^6 +\displaystyle \sum _{4\le 4}m_{44}(\Omega ) \quad g^4 h^4 +\displaystyle \sum _{4\le 5}m_{45}(\Omega ) \quad g^4 h^5\\ {}&+\displaystyle \sum _{4\le 6}m_{46}(\Omega ) \quad g^4 h^6 +\displaystyle \sum _{5\le 5}m_{55}(\Omega ) \quad g^5 h^5 +\displaystyle \sum _{5\le 6}m_{56}(\Omega ) \quad g^5 h^6\\ =&|\lambda _{(3,3)}|\quad g^3 h^3 +|\lambda _{(3,4)}|\quad g^3 h^4 +|\lambda _{(3,5)}|\quad g^3 h^5\\ {}&+|\lambda _{(3,6)}|\quad g^3 h^6 +|\lambda _{(4,4)}|\quad g^4 h^4 +|\lambda _{(4,5)}|\quad g^4 h^5\\ {}&+|\lambda _{(4,6)}|\quad g^4 h^6 +|\lambda _{(5,5)}|\quad g^5 h^5 +|\lambda _{(5,6)}|\quad g^5 h^6\\\\ =&(2r+4)g^3 h^3 +(4r-4)g^3 h^4 +(4c-4)g^3 h^5 +(4r+2c) g^3 h^6 +(cr-c)g^4 h^4\\ {}&+4(r-1)(c-1)g^4 h^5 +(2cr-2c)g^4 h^6 +(cr-r)g^5 h^5 +(4cr-4r)g^5 h^6 \end{aligned}$$

Hence the proof. $\square$

### M-Polynomial of a chemical graph

In this section, a program code using Java programming language was developed, that enables us to generate the M-polynomial of any chemical graph. The program code is as follows.

**Program Code:**

var totalinput = "";

var inputx = "";

var inputy = "";

var inputequ = "";

var inputequ = document.getElementById("inputequ");

var inputx = document.getElementById("inputx");

var inputy = document.getElementById("inputy"); var inputbutton = document.getElementById("addquation");

var resultcontainer = document.getElementById("result");

window.addEventListener("DOMContentLoaded", (event) => {

inputbutton.addEventListener("click", myFunction);

});

function myFunction() {

var xval = inputx.value;

var yval = inputy.value;

var eqval = inputequ.value;

//console.log(resultcontainer.innerHTML); if (resultcontainer.innerHTML != ""){

resultcontainer.innerHTML += " + ";

}

resultcontainer.innerHTML += "(" + eqval + ") * g<sup>"+xval+"</sup> * h<sup>"+yval+"</sup>";

}

**Output Window:**

**Figure 3:**
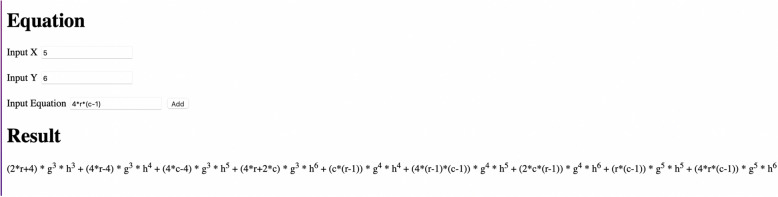
The output window for the given program code for generating M-polynomial.

Once the program is runned, the output window as given in Fig. 3 is obtained. In this window, the edge partition data of the chemical structure is needed to be inputed inorder to obtain the M-polynomial. Suppose there are *x* number of edges with end vertex degree $(d_{\mu },d_{\eta })$, then in ‘Input X’ column, we have to input the value of $d_{\mu }$, in ‘Input Y’ column, we have to input the value of $d_{\eta }$ and in ‘Input Equation’ column, we have to input the value of *x*. Next, the inputed data is added by clicking the ‘Add’ button. Likewise, each data is added and as a final outcome, the required M-polynomial equation is obtained.

## Degree based descriptors of $\beta _{12}$: borophene nanosheet

### Results based on M-polynomial approach

This section comprises of theorems based on the derivation of degree based descriptors of $\beta _{12}$ - borophene nanosheet, $\beta _{12} - BN(r,c); r, c\ge 1$ using M-polynomial.

#### Theorem 2

For $$G=\beta _{12} - BN(r,c); r, c\ge 1$$

$$\begin{aligned}&M_1(G)=118\quad cr - 14\quad r - 14\quad c\\&M_2(G)=289\quad cr - 87\quad r - 48\quad c + 8\\&M_2^m(G)=\frac{623 \quad cr}{1200} +\frac{91\quad r}{225} +\frac{23\quad c}{720} + \frac{2}{45}\\&H(G)= \frac{4883 \quad cr }{1980} + \frac{1019 \quad r}{1155} - \frac{17\quad c}{180} + \frac{5}{63}. \end{aligned}$$

#### Proof

The M-polynomial of $\Omega =\beta _{12} - BN(r,c)$ is,

$$\begin{aligned} M(\Omega ;g,h) =\,& (2r+4)g^3 h^3 +(4r-4)g^3 h^4 +(4c-4)g^3 h^5 +(4r+2c) g^3 h^6 +(cr-c)g^4 h^4\\ {}&+4(r-1)(c-1)g^4 h^5 +(2cr-2c)g^4 h^6 +(cr-r)g^5 h^5 +(4cr-4r)g^5 h^6. \end{aligned}$$

Let $$t(g,h) = M(\Omega ;g,h)$$. Then,

$$\begin{aligned}(D_g+D_h)(t(g,h))&=2\, g^3 h^3 \, \big (6\, r - 14\, h + 14\, hr + 16\, c\, h^2 + 9\, c\, h^3 + 18\, g\, h^2 + 18\, r \, h^3 - 16\, h^2 \\ {}&\quad - 5\, r\, g^2 h^2 - 22\, r \, g^2 h^3 - 4\, c\, g h - 18\, c\, g \, h^2 - 10\, c\, g\, h^3 - 18\, r \, g\, h^2 + 4\, cr\, gh \\ {}&\quad + 18\, cr \, g\, h^2 + 10\, cr\, g\, h^3 + 5\, cr\, g^2 h^2 + 22\, cr\, g^2 h^3 + 12\big ) \\ D_g D_h(t(g,h))&=g\, h\, \big (15 g^2 h^4 (4c - 4) + 9 g^2 h^2 (2r + 4) + 12 g^2 h^3 (4r - 4) + 18 g^2 h^5 (2c + 4r) \\ {}&\quad + 16 g^3 h^3 (cr - c) + 48 g^3 h^5 (cr - c) + 25 g^4 h^4 (rc - r) + 120 g^4 h^5 (rc - r) \\&\quad + 20 g^3 h^4 (4 r - 4)(c - 1)\big ) \\ S_g S_h(t(g,h))&=\frac{g^3 h^5 (4c - 4)}{15} + \frac{g^3 h^3 (2 r + 4)}{9} + \frac{g^3 h^4 (4 r - 4)}{12} - \frac{g^4 h^6 (2 c - 2cr)}{24} \\&\quad - \frac{g^5 h^6 (4r - 4cr)}{30} + \frac{g^3 h^6 (2 c + 4 r)}{18} - \frac{g^4 h^4 (c - cr)}{16} - \frac{ g^5 h^5 (r - cr)}{25} \\&\quad + \frac{g^4 h^5 (4 r - 4)(c - 1)}{20} \end{aligned}$$

$$\begin{aligned} 2 S_g J(t(g,h))&=2 \bigg (\frac{ g^9(2c + 4r)}{9} + \frac{g^8 (c - 1)}{2} + \frac{g^6 (r + 2)}{3} + \frac{g^7 (4r - 4)}{7} - \frac{g^{10} (c - cr)}{5} \\&\quad - \frac{g^8 (c - cr)}{8} - \frac{g^{10} (r - cr)}{10} - \frac{g^{11} (4r - 4cr)}{11} + \frac{g^9 (4r - 4)(c - 1)}{9}\bigg ). \end{aligned}$$

By using Table 1,

$$\begin{aligned} M_1(\Omega )&=(D_g+D_h)(t(g,h))|_{g=h=1}\\& =118\; cr - 14\; r - 14\; c \\ M_2(\Omega )&=D_g D_h(t(g,h))|_{g=h=1}\\& =289\; cr - 87\; r - 48\; c + 8 \\ M_2^m(\Omega )&=S_g S_h(t(g,h))|_{g=h=1}\\& =\frac{623 \; cr}{1200} +\frac{91\; r}{225} +\frac{23\; c}{720} + \frac{2}{45} \\ H(\Omega )&=2 S_g J(t(g,h))|_{g=h=1}\\& =\frac{4883 \; cr }{1980} + \frac{1019 \; r}{1155} - \frac{17\; c}{180} + \frac{5}{63}. \end{aligned}$$

#### Theorem 3

For $$\Omega =\beta _{12} - BN(r,c); r, c\ge 1$$

$$\begin{aligned} R_{\kappa }(G)&=18^{\kappa }(2c + 4r) + 15^{\kappa }(4c - 4) + 12^{\kappa }(4r - 4) + 3^{2\kappa }(2r + 4) + 20^{\kappa }(4r - 4)(c - 1) \\&\quad + 2^{4\kappa }(cr - c) + 5^{2\kappa }(rc - r) + 2^{\kappa +2} \quad 15^{\kappa }(rc - r) + 2^{3\kappa + 1} \quad 3^{\kappa }(cr - c) \\ RR_{\kappa }(\Omega )&=\frac{1}{18^{\kappa }}(2c + 4r) + \frac{1}{15^{\kappa }}(4c - 4) + \frac{1}{3^{2\kappa }}(2r + 4) + \frac{1}{12^{\kappa }}(4r - 4) + \frac{1}{20^{\kappa }}(4r - 4)(c - 1) \\&\quad + \frac{1}{2^{4\kappa }}(cr - c) + \frac{1}{5^{2\kappa }}(rc - r) + \frac{2^{1 - 3\kappa }}{3^{\kappa }} (cr - c) + \frac{2^{2 - \kappa }}{15^{\kappa }}(rc - r). \end{aligned}$$

#### Proof

The proof is similar to Theorem 2. Let $t(g,h) = M(\Omega ;g,h)$, then

$$\begin{aligned} R_{\kappa }(\Omega )&=D_g^{\kappa } D_h^{\kappa }(t(g,h))|_{g=h=1}\\&\quad =18^{\kappa }(2c + 4r) + 15^{\kappa }(4c - 4) + 12^{\kappa }(4r - 4) + 3^{2\kappa }(2r + 4) + 20^{\kappa }(4r - 4)(c - 1) \\&\quad + 2^{4\kappa }(cr - c) + 5^{2\kappa }(rc - r) + 2^{\kappa +2} \quad 15^{\kappa }(rc - r) + 2^{3\kappa + 1} \quad 3^{\kappa }(cr - c) \\ RR_{\kappa }(\Omega )&=S_g^{\kappa } S_h^{\kappa }(t(g,h))|_{g=h=1}\\&\quad =\frac{1}{18^{\kappa }}(2c + 4r) + \frac{1}{15^{\kappa }}(4c - 4) + \frac{1}{3^{2\kappa }}(2r + 4) + \frac{1}{12^{\kappa }}(4r - 4) + \frac{1}{20^{\kappa }}(4r - 4)(c - 1) \\&\quad + \frac{1}{2^{4\kappa }}(cr - c) + \frac{1}{5^{2\kappa }}(rc - r) + \frac{2^{1 - 3\kappa }}{3^{\kappa }} (cr - c) + \frac{2^{2 - \kappa }}{15^{\kappa }}(rc - r). \end{aligned}$$

#### Theorem 4

For $$\Omega =\beta _{12} - BN(r,c); r, c\ge 1$$

$$\begin{aligned} HM(\Omega )&=1172 \quad cr - 316 \quad r - 170 \quad c + 16\\ F(\Omega )&=594 \quad cr - 142 \quad r - 74 \quad c\\ \sigma (\Omega )&=16 \quad rc + 32 \quad r +22 \quad c - 16. \end{aligned}$$

#### Proof

Let $t(g,h) = M(\Omega ;g,h)$, then by using the Table 1,

$$\begin{aligned} HM(\Omega )&=(D_g+D_h)^2(t(g,h))|_{g=h=1}\\&=1172 \quad cr - 316 \quad r - 170 \quad c + 16 \\ F(\Omega )&=(D_g^2 + D_h^2)(t(g,h))|_{g=h=1}\\&=594 \quad cr - 142 \quad r - 74 \quad c \\ \sigma (\Omega )&=(D_g - D_h)^2(t(g,h))|_{g=h=1}\\&=16 \quad rc + 32 \quad r +22 \quad c - 16. \end{aligned}$$

#### Theorem 5

For $$\Omega =\beta _{12} - BN(r,c); r, c\ge 1$$

$$\begin{aligned} SDD(\Omega )&= \frac{74 \; cr}{3} +4 \; r - \frac{7\; c}{15} - \frac{6}{5}\\ I(\Omega )&=\frac{28,807 \; cr}{990} - \frac{6155 \; r}{1386} - \frac{377 \; c}{90} + \frac{67}{126}\\ A(\Omega )&=\frac{545,166,215 \; cr}{1,580,544} - \frac{74,604,408,241 \; r}{592,704,000} - \frac{1,291,939 \; c}{18522} + \frac{14,447,819}{686,000}. \end{aligned}$$

#### Proof

Let $t(g,h) = M(\Omega ;g,h)$. Then by using Table 1,

$$\begin{aligned} SDD(\Omega )&=(D_g S_h+ D_h S_g)(t(g,h))|_{g=h=1}\\&=\frac{74 \; cr}{3}+4 \; r - \frac{7\; c}{15} - \frac{6}{5} \\ I(\Omega )&=S_g J D_g D_h(t(g,h))|_{g=h=1}\\&=\frac{28807 \; cr}{990} - \frac{6155 \; r}{1386} - \frac{377 \; c}{90} + \frac{67}{126} \\ A(\Omega )&=S_g^3 Q_{-2} J D_g^3 D_h^3(t(g,h))|_{g=h=1}\\&=\frac{545,166,215 \; cr}{1,580,544} - \frac{74,604,408,241 \; r}{592,704,000} - \frac{1,291,939 \; c}{18522} + \frac{14,447,819}{686,000}. \end{aligned}$$

$$\square$$

### Results based on edge partition technique

This section comprises of theorems based on the computation of degree based descriptors of $\beta _{12}$—borophene nanosheet, $\beta _{12} - BN(r,c); r, c\ge 1$. In this section, the degree counting method and edge partition technique is applied and the expressions are derived from the definition of the corresponding degree based descriptors.

#### Theorem 6

For $$\Omega =\beta _{12}-BN(r,c); r, c\ge 1$$

$$\begin{aligned} ABC(\Omega )&=\frac{4 \; r}{3} + \frac{\sqrt{14}\; (c + 2r)}{3} +\frac{\sqrt{10} \; (4c - 4)}{5} + \frac{\sqrt{15} \; (2 r - 2)}{3} + \frac{\sqrt{35}\; (4r - 4) (c - 1)}{10} \\&\quad + \frac{2\sqrt{2}\; (rc - r)}{5} + \frac{2\sqrt{3}\; (cr - c)}{3} + \frac{\sqrt{6} \; (cr - c)}{4} + \frac{2\sqrt{30}\; (rc - r)}{5} + \frac{8}{3} \\ GA(\Omega )&=2 \; cr + r - c \; - \; \frac{2\sqrt{6} \; (2c - 2cr)}{5} - \frac{2\sqrt{30} \; (4 r - 4cr)}{11} + \frac{2\sqrt{2}\; (2c + 4r)}{3} \\&\; + \frac{\sqrt{15} \; (4c - 4)}{4} + \frac{4\sqrt{3}\; (4r - 4)}{7} + \frac{4\sqrt{5} \; (4r - 4)(c - 1)}{9} + 4 \\ SC(\Omega )&=\frac{3\sqrt{2} \; c}{4} - \frac{2\; c}{3} - \frac{\sqrt{10} \; c}{5} + \frac{\sqrt{6} \; r}{3} + \frac{4\sqrt{7} \; r}{7} - \frac{\sqrt{10} \; r}{10} - \frac{4\sqrt{11} \; r}{11} + \frac{4 \; cr}{3} - \sqrt{2} \\&\quad + \frac{2\sqrt{6}}{3} - \frac{4\sqrt{7}}{7} + \frac{\sqrt{2} \; cr}{4} + \frac{3\sqrt{10} \; cr}{10} + \frac{4\sqrt{11} \; cr}{11} + \frac{4}{3}. \end{aligned}$$

#### Proof

Let $\Omega =\beta _{12}-BN(r,c); r, c\ge 1$. $\Omega$ has $5rc+2r$ vertices and $(12r-1)c+r$ edges. The edge set partitions based on the vertex degree are $\lambda _{(3,3)}$, $\lambda _{(3,4)}$, $\lambda _{(3,5)}$, $\lambda _{(3,6)}$, $\lambda _{(4,4)}$, $\lambda _{(4,5)}$, $\lambda _{(4,6)}$, $\lambda _{(5,5)}$ and $\lambda _{(5,6)}$ for *G*, where, $\lambda _{(i,j)}=\{\mu \eta \in E(G): d_{\mu }=i, d_{\eta }=j\}$. Also,

$$|\lambda _{(3,3)}|=2r+4$$; $$|\lambda _{(3,4)}|=4r-4$$; $$|\lambda _{(3,5)}|=4c-4$$; $$|\lambda _{(3,6)}|=4r+2c$$; $$|\lambda _{(4,4)}|=rc-c$$;

$$|\lambda _{(4,5)}|=4(r-1)(c-1)$$; $$|\lambda _{(4,6)}|=2c(r-1)$$; $$|\lambda _{(5,5)}|=rc-r$$; $$|\lambda _{(5,6)}|=4r(c-1)$$.

By definition, Atom bond connectivity index, $ABC(\Omega )=\displaystyle \sum _{\mu \eta \in E(\Omega )}\frac{\sqrt{d_{\mu }+d_{\eta } -2}}{\sqrt{d_{\mu } \times d_{\eta }}}$

$$\begin{aligned} ABC(\Omega ) =\,& |\lambda _{(3,3)}| \times \frac{2}{3} +|\lambda _{(3,4)}| \times \frac{\sqrt{5}}{\sqrt{12}} +|\lambda _{(3,5)}| \times \frac{\sqrt{2}}{\sqrt{5}} +|\lambda _{(3,6)}| \times \frac{\sqrt{7}}{\sqrt{18}} +|\lambda _{(4,4)}| \times \frac{\sqrt{6}}{4}\\ {}&+|\lambda _{(4,5)}| \times \frac{\sqrt{7}}{\sqrt{20}} +|\lambda _{(4,6)}| \times \frac{1}{\sqrt{3}} +|\lambda _{(5,5)}| \times \frac{\sqrt{8}}{5} +|\lambda _{(5,6)}| \times \frac{\sqrt{9}}{\sqrt{30}}\\ =\,& \frac{4 \; r}{3} + \frac{\sqrt{14}\; (c + 2r)}{3} +\frac{\sqrt{10} \; (4c - 4)}{5} + \frac{\sqrt{15} \; (2 r - 2)}{3} + \frac{\sqrt{35}\; (4r - 4) (c - 1)}{10} \\ {}&+ \frac{2\sqrt{2}\; (rc - r)}{5} + \frac{2\sqrt{3}\; (cr - c)}{3} + \frac{\sqrt{6} \; (cr - c)}{4} + \frac{2\sqrt{30}\; (rc - r)}{5} + \frac{8}{3}. \end{aligned}$$

Geometric arithmetic index, $GA(\Omega )=\displaystyle \sum _{\mu \eta \in E(\Omega )}\frac{2\sqrt{d_{\mu } \times d_{\eta }}}{d_{\mu } + d_{\eta }}$

$$\begin{aligned} GA(\Omega )=\, &|\lambda _{(3,3)}| \times 1 +|\lambda _{(3,4)}| \times \frac{4\sqrt{3}}{7} +|\lambda _{(3,5)}| \times \frac{\sqrt{15}}{4} +|\lambda _{(3,6)}| \times \frac{2\sqrt{2}}{3} +|\lambda _{(4,4)}| \times 1\\ {}&+|\lambda _{(4,5)}| \times \frac{4\sqrt{5}}{9} +|\lambda _{(4,6)}| \times \frac{2\sqrt{6}}{5} +|\lambda _{(5,5)}| \times 1 +|\lambda _{(5,6)}| \times \frac{2\sqrt{30}}{11}\\ =\, &2 \; cr + r - c \; - \; \frac{2\sqrt{6} \; (2c - 2cr)}{5} - \frac{2\sqrt{30} \; (4 r - 4cr)}{11} + \frac{2\sqrt{2}\; (2c + 4r)}{3} \\ {}&+ \frac{\sqrt{15} \; (4c - 4)}{4} + \frac{4\sqrt{3}\; (4r - 4)}{7} + \frac{4\sqrt{5} \; (4r - 4)(c - 1)}{9} + 4. \end{aligned}$$

Sum connectivity index, $SC(\Omega )=\displaystyle \sum _{\mu \eta \in E(\Omega )}\frac{1}{\sqrt{d_{\mu }+d_{\eta }}}$

$$\begin{aligned} SC(\Omega )=\, &|\lambda _{(3,3)}| \times \frac{1}{\sqrt{6}} +|\lambda _{(3,4)}| \times \frac{1}{\sqrt{7}} +|\lambda _{(3,5)}| \times \frac{1}{\sqrt{8}} +|\lambda _{(3,6)}| \times \frac{1}{3} +|\lambda _{(4,4)}| \times \frac{1}{\sqrt{8}}\\ {}&+|\lambda _{(4,5)}| \times \frac{1}{3} +|\lambda _{(4,6)}| \times \frac{1}{\sqrt{10}} +|\lambda _{(5,5)}| \times \frac{1}{\sqrt{10}} +|\lambda _{(5,6)}| \times \frac{1}{\sqrt{11}}\\\\ =\, &\frac{3\sqrt{2} \; c}{4} - \frac{2\; c}{3} - \frac{\sqrt{10} \; c}{5} + \frac{\sqrt{6} \; r}{3} + \frac{4\sqrt{7} \; r}{7} - \frac{\sqrt{10} \; r}{10} - \frac{4\sqrt{11} \; r}{11} + \frac{4 \; cr}{3} - \sqrt{2} \\ {}&+ \frac{2\sqrt{6}}{3} - \frac{4\sqrt{7}}{7} + \frac{\sqrt{2} \; cr}{4} + \frac{3\sqrt{10} \; cr}{10} + \frac{4\sqrt{11} \; cr}{11} + \frac{4}{3}. \end{aligned}$$

$$\square$$

## Discussion

**Figure 4:**
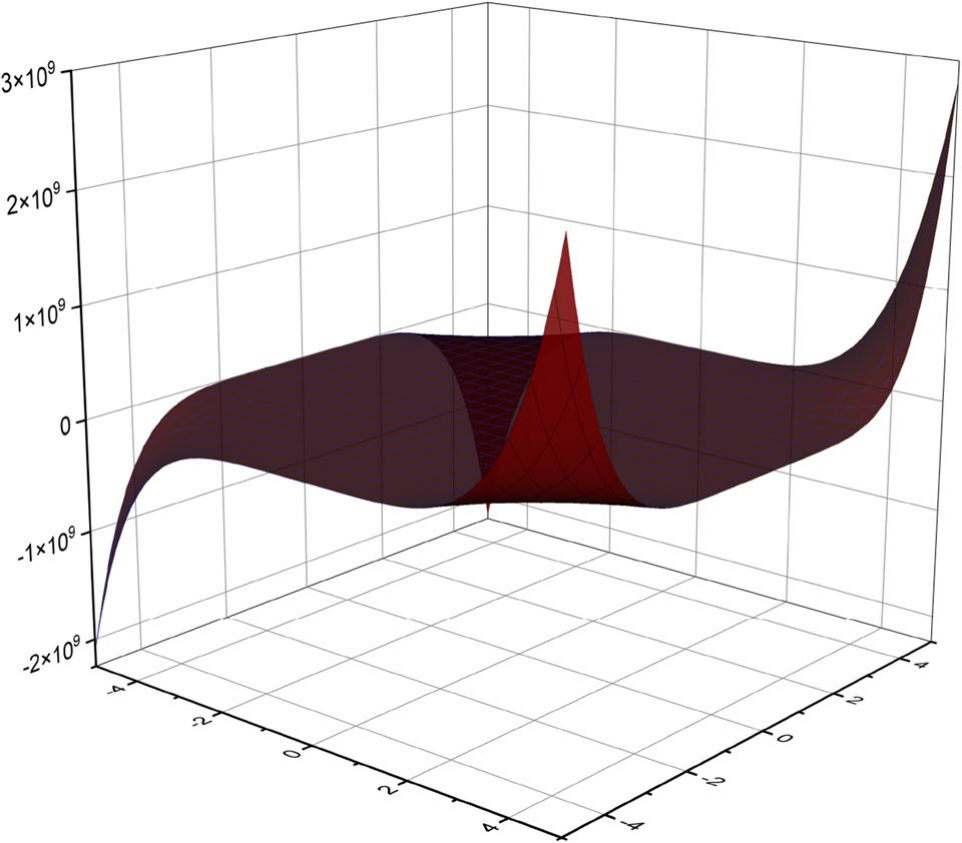
Graphical representation of M-polynomial for the nanosheet, $\beta _{12}-BN(4,4)$.

**Figure 5:**
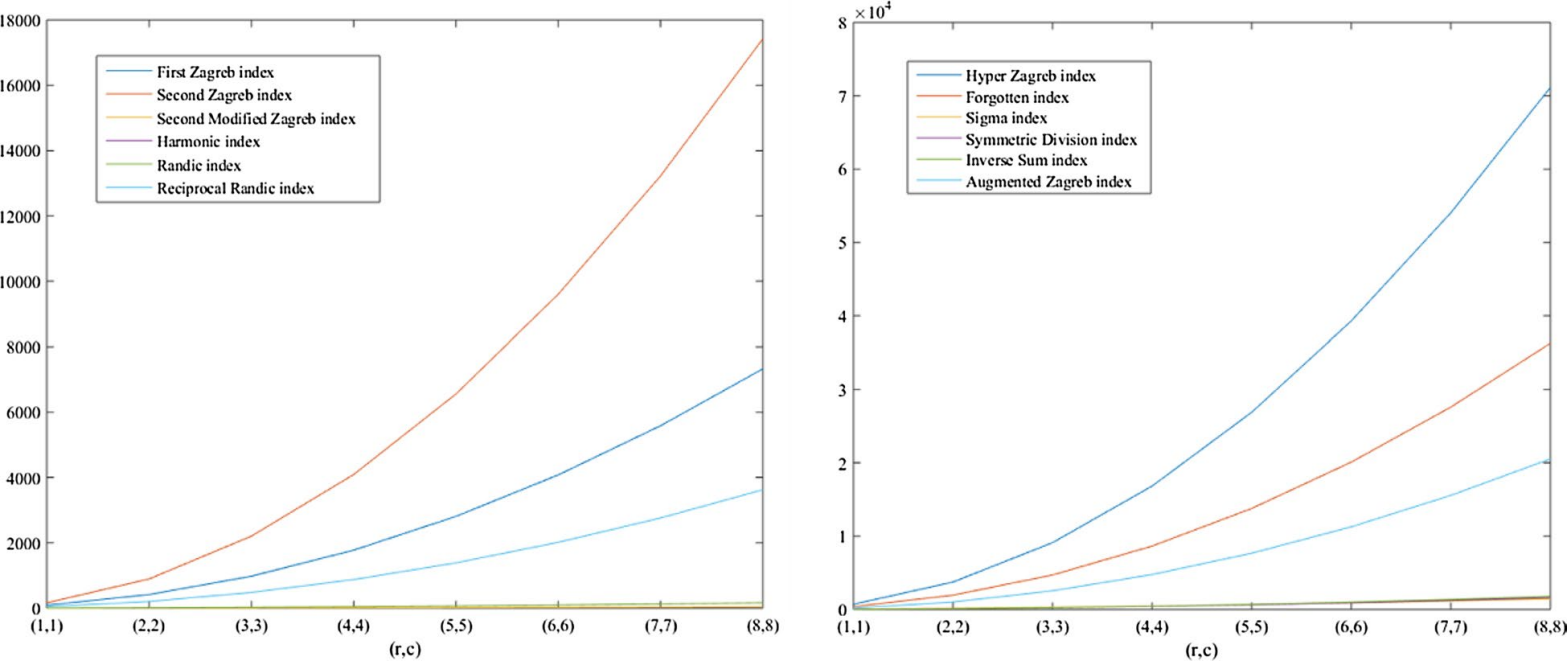
Comparison graph based on the descriptors of $\beta _{12}$-borophene nanosheet.

**Figure 6:**
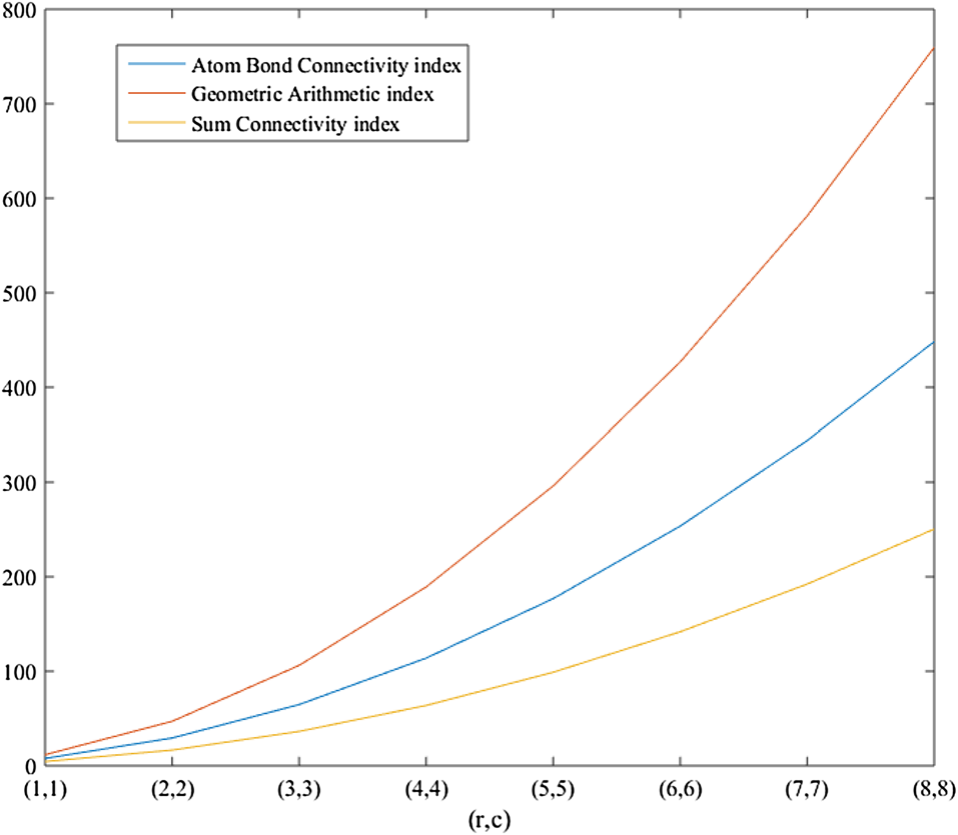
Comparison graph based on the descriptors of $\beta _{12}$-borophene nanosheet.

In the previous section, the degree based descriptors of $\beta _{12}$-Borophene nanosheet were derived from the M-polynomial of the graph. Using those analytic expressions, the numerical values of the descriptors for some fixed value of the parameters *r* and *c* of $\beta _{12}-BN(r,c)$ were computed and are presented in Tables 2 and 3. The graph plotted using the M-polynomial function of the nanosheet is depicted in Fig. 4. The numerical comparison of degree based descriptors computed in Theorem 6 between some fixed values of the parameters *r* and *c* are shown in Table 4. The numerical computations was done with the help of MATLAB sofware. The computed numerical values were plotted inorder to obtain a graphical comparison between the descritors and parameters *r* and *c*, and the same are exhibited in Figs. 5 and 6.

**Table 2:** Computed numerical values of degree based descriptors for $\beta _{12}-BN(r,c)$.

(*r*, *c*)	(1,1)	(2,2)	(3,3)	(4,4)	(5,5)	(6,6)
First Zagreb	90	416	978	1776	2810	4080
Second Zagreb	162	894	2204	4092	6558	9602
Second modified Zagreb	1	2.9939	6.0261	10.0967	15.2056	21.3528
Harmonic	3.3333	11.5196	24.6382	42.6892	65.6724	93.5880
Randić	3.4142	11.7541	25.0599	43.3317	66.5694	94.7731
Reciprocal Randić	43.4558	203.5623	480.8595	875.3476	1387.0265	2015.8962

**Table 3:** Computed numerical values of degree based descriptors for $\beta _{12}-BN(r,c)$.

(*r*, *c*)	(1,1)	(2,2)	(3,3)	(4,4)	(5,5)	(6,6)
Hyper Zagreb	702	3732	9106	16,824	26,886	39,292
Forgotten	378	1944	4698	8640	13,770	20,088
Sigma	54	156	290	456	654	884
Symmetric division	27	104.5	231.4	407.6	633.1	908
Inverse sum	21	99.6642	236.5244	431.5805	684.8326	996.2807
Augmented Zagreb	170.3612	1009.5078	2538.5007	4757.3399	7666.0253	11,264.5571

**Table 4:** Computed numerical values of degree based descriptors for $\beta _{12}-BN(r,c)$.

(*r*, *c*)	(1,1)	(2,2)	(3,3)	(4,4)	(5,5)	(6,6)
Atom bond connectivity	7.7416	29.3422	64.7228	113.8837	176.8247	253.5459
Geometric arithmetic	11.6568	47.0069	106.1935	189.2167	296.0764	426.7725
Sum connectivity	4.4495	16.5419	36.3176	63.7766	98.9187	141.7441

## Quantitative structure-activity-property relationship model

Theoretical topological descriptors are numerical representations of chemical compounds that encode the topological and chemical information of the structure. A variety of descriptors, such as physicochemical, constitutional and geometrical, electrostatic, topological and quantum chemical indices, have been developed and are widely used in quantitative structure-activity-property research to predict biological activities and chemical properties. The process begins with a suitable molecular topological descriptor and ends with some inference, hypothesis, and prediction about the molecule’s behaviour, properties and characteristics. The availability of high-quality experimental data, as well as its correctness, are vital in this investigation. The selection of the optimal topological descriptor for modelling in analysis is a crucial phase in the QSPR/QSAR process. Because there is no consensus on the best molecular description, all feasible descriptors are determined. A major application of QSPR/QSAR models is that the properties, activities, behavior, etc. of a newly designed or untested chemical compound can be inferred from the molecular structure of similar compounds whose properties, activities, characteristics, etc. have already been assessed. Recently researchers are coming up with relevant results in this field of research.

**Figure 7:**
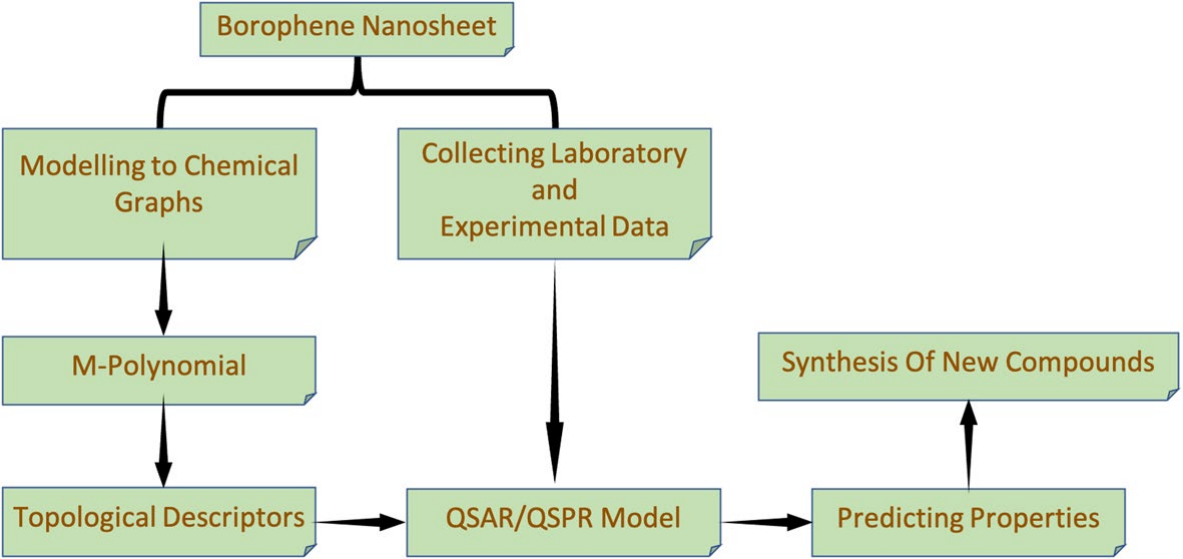
QSAR/QSPR flow chart model.

A flow chart diagram is drawn based on the QSPR/QSAR modelling with respect to structural descriptors of chemical structures, especially with respect to the borophene nanosheet. The same is presented in Figure 7.

### Linear regression model

As mentioned, $2-Pmmn$, $\beta _{12}$, $\chi ^3$ are the most researched borophene nanostructures. In this section, the linear regression models for few properties of these borophene nanostructures with their degree based descriptors are obtained using least square procedure.

The following regression model is considered for the analysis.

$$\begin{aligned} P= x (SD) + y, \end{aligned}$$

where *P* and *SD* stands for property and structural descriptor respectively.

For the study, the properties such as shear modulus (*G*), Young’s modulus (*Y*) and Poisson’s ratio $(\nu )$ are considered. The numerical values of various topological descriptors and the properties^39^ for corresponding borophene nanostuctures are given in Tables 5 and 6 respectively.

**Table 5:** Numerical values of degree based descriptors for the borophene structures.

Descriptor	$2-Pmmn$	$\beta _{12}$	$\chi ^3$
First Zagreb	90	416	978
Second Zagreb	162	894	2204
Second modified Zagreb	1	2.9939	6.0261
Harmonic	3.333	11.5196	24.6382
Hyper Zagreb	702	3732	9106
Forgotten	378	1944	4698
Sigma	54	156	290
Symmetric division	27	104.5333	231.4
Inverse sum	21	99.6642	236.5244
Augmented Zagreb	170.361	1009.5078	2538.5007
Atom bond connectivity	7.7416	29.342	64.7228
Geometric arithmetic	11.6568	47.0069	106.1935
Sum connectivity	11.6568	47.0069	106.1935

**Table 6:** Values of the properties for the borophene structures.

Descriptor	$2-Pmmn$	$\beta _{12}$	$\chi ^3$
Shear modulus	94	68.5	60.5
Young’s modulus	398	179	198.5
Poisson’s ratio	− 0.04	0.176	0.116

Using the Microsoft Excel analytical tools, the regression analysis was carried out and the correlation coefficients ($\mathcal {R}$) generated are given in Tables 7 and 8.

**Table 7:** Correlation coefficients ($\mathcal {R}$) between the properties and degree based descriptors of the borophene structures.

Descriptor	Shear modulus	Young’s modulus	Poisson’s ratio
First Zagreb	0.76	0.93	0.98
Second Zagreb	0.95	0.9999	0.97
Second modified Zagreb	0.89	0.71	0.56
Harmonic	0.79	0.56	0.39
Hyper Zagreb	0.96	0.9994	0.97
Forgotten	0.97	0.9983	0.96
Sigma	0.05	0.35	0.52
Symmetric division	0.36	0.06	0.13
Inverse sum	0.75	0.91	0.97
Augmented Zagreb	0.95	0.9999	0.97

**Table 8:** Correlation coefficients ($\mathcal {R}$) between the properties and degree based descriptors of the borophene structures.

Descriptor	Shear modulus	Young’s modulus	Poisson’s ratio
Atom bond connectivity	0.60	0.33	0.14
Geometric arithmetic	0.29	0.01	0.2
Sum connectivity	0.65	0.39	0.2

**Figure 8:**
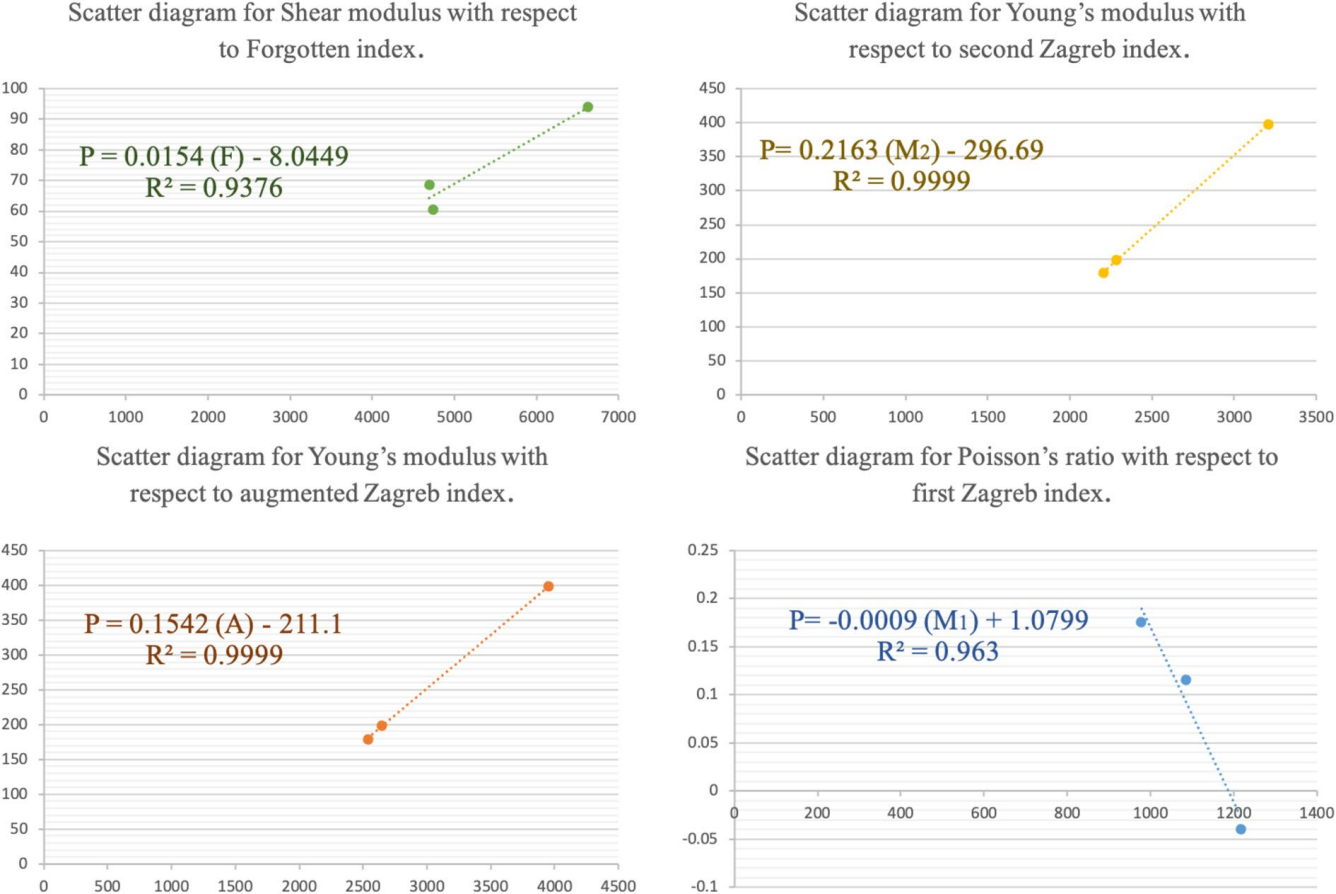
Scatter diagram showing relation between the property and the descriptor.

Based on the correlation coefficient ($\mathcal {R}$), the regression model for Forgotten index, (*F*) is found to be best fitted for the shear modulus, and the regression equation can be given as

$$\begin{aligned} P=0.0154 (F) - 8.0449. \end{aligned}$$

For Young’s modulus, the regression model for both second Zagreb, $(M_2)$ and augmented Zagreb index, (*A*) is found to have same correlation coefficent.

The regression equation with respect to first Zagreb index can be given as

$$\begin{aligned} P=0.2163 (M_2) - 296.69. \end{aligned}$$

The regression equation with respect to augmented Zagreb index can be given as

$$\begin{aligned} P=0.1542(A) - 211.1. \end{aligned}$$

For Poisson’s ratio, the regression model for first Zagreb index, $(M_1)$ is found to be best fitted and the regression equation can be given as

$$\begin{aligned} P=-0.0009 (M_1) + 1.0799. \end{aligned}$$

The scatter diagrams that show relation between properties and their best fitted descriptors are given in Fig. 8.

Thus it can be concluded that, the above mentioned properties of different variations of borophene nanostructures can be predicted with the help of regression equations using the computed topological descriptors.

## Conclusion

In this work, the degree based structural descriptors of $\beta _{12}$-borophene nanosheet were computed using the M-polynomial approach. That degree based structural descriptors can be routinely computed from the M-polynomial. This helps in reducing the problem of determining the expression in each particular case to a single problem of determining the M-polynomial, thus making the process general, fast and efficient. Our work is reduced to determining the edge partitions due to the developed program code’s relative ease of use. The descriptors is having application in disparate fields and thus the study is relevant in present scenario. The analytic expressions derived and the numerical and graphical comparison together with the Quantitative Structure-Activity-Property Relationship model may help researchers and scientists in future advancements in the field of nanomaterials and to study the nature and behaviour of the $\beta _{12}$-borophene nanosheet and other borophene nanostructures without any laboratory experimentations.

## Data Availability

All data generated or analysed during this study are included in this published article.
