# Author Correction: A novel perspective for M-polynomials to compute molecular descriptors of borophene nanosheet

**DOI:** 10.1038/s41598-023-40229-y

**Published:** 2023-08-10

**Authors:** Rashad Ismail, Annmaria Baby, D. Antony Xavier, Eddith Sarah Varghese, Muhammad Usman Ghani, A. Theertha Nair, Hanen Karamti

**Affiliations:** 1 Department of Mathematics, Faculty of Science and Arts, Muhayl Assir, King Khalid University, Abha, Saudi Arabia; 2 Department of Mathematics, Loyola College, University of Madras, Chennai, India; 3 Institute of Mathematics, Khawaja Fareed University of Engineering & Information Technology, Abu Dhabi Road, 64200 Rahim Yar Khan, Pakistan; 4 Department of Computer Sciences, College of Computer and Information Sciences, Princess Nourah bint Abdulrahman University, P.O.Box 84428, Riyadh, 11671 Saudi Arabia

Correction to: *Scientific Reports* https://doi.org/10.1038/s41598-023-37637-5, published online 25 July 2023

The Acknowledgements section in the original version of this Article was incomplete. It now reads:

The authors extend their appreciation to the Deanship of Scientific Research at King Khalid University for funding this work through a large group Research Project under grant number (R.G.P.2/163/44). Princess Nourah bint Abdulrahman University Researchers Supporting Project number (PNURSP2023R192), Princess Nourah bint Abdulrahman University, Riyadh, Saudi Arabia.

The original Article has been corrected.

